# Detection of antimicrobial resistance genes associated with the International Space Station environmental surfaces

**DOI:** 10.1038/s41598-017-18506-4

**Published:** 2018-01-16

**Authors:** C. Urbaniak, A. Checinska Sielaff, K. G. Frey, J. E. Allen, N. Singh, C. Jaing, K. Wheeler, K. Venkateswaran

**Affiliations:** 1 Jet Propulsion Laboratory, California Institute of Technology, Pasadena, CA USA; 2 Naval Medical Research Center-Frederick, Frederick, MD USA; 3 Lawrence Livermore National Laboratory, Livermore, CA USA; 4 Allosource, Centennial, CO USA; 5 Present Address: Department of Ecology, Evolution, and Organismal Biology, Iowa State University, Ames, IA USA

## Abstract

Antimicrobial resistance (AMR) is a global health issue. In an effort to minimize this threat to astronauts, who may be immunocompromised and thus at a greater risk of infection from antimicrobial resistant pathogens, a comprehensive study of the ISS “resistome’ was conducted. Using whole genome sequencing (WGS) and disc diffusion antibiotic resistance assays, 9 biosafety level 2 organisms isolated from the ISS were assessed for their antibiotic resistance. Molecular analysis of AMR genes from 24 surface samples collected from the ISS during 3 different sampling events over a span of a year were analyzed with Ion AmpliSeq^™^ and metagenomics. Disc diffusion assays showed that *Enterobacter bugandensis* strains were resistant to all 9 antibiotics tested and *Staphylococcus haemolyticus* being resistant to none. Ion AmpliSeq^™^ revealed that 123 AMR genes were found, with those responsible for beta-lactam and trimethoprim resistance being the most abundant and widespread. Using a variety of methods, the genes involved in antimicrobial resistance have been examined for the first time from the ISS. This information could lead to mitigation strategies to maintain astronaut health during long duration space missions when return to Earth for treatment is not possible.

## Introduction

The International Space Station (ISS) is not a sterile environment. Microbial monitoring of air and surfaces have shown that many different microbes exist in this closed, harsh environment, some of which are opportunistic pathogens of human origin^1–4^. The presence of such microorganisms within this environment poses a serious risk to crew health, as opportunistic pathogens have shown increased virulence while grown in space and prolonged spaceflight weakens an astronaut’s immune system, rendering him/her more susceptible to infection^5^. Some human associated opportunistic pathogens that have been isolated from the ISS include *Staphylococcus aureus*, *S. haemolyticus*, *S. hominis*, *Acinetobacter pitti*, *Pantoea conspicua* and members of the family *Enterobacteriaceae*, while others such as *Propionibacterium* and *Corynebacterium* have been detected with culture-independent, molecular based methods^2,5^. This study however, does not discuss virulence – a key factor that determines whether or not a microbe could be harmful to humans. Thus, the AMR gene carrying bacteria reported in this study do not necessarily pose a threat to astronauts on the ISS.

Antibiotics are the primary treatment strategy to combat infections, but this approach may not be as easy to implement on the ISS or on future deep space missions. Studies have shown that some organisms become less susceptible to antibiotic action when exposed to spaceflight. For example, the minimum inhibitory concentration (MIC) of colistin and kanamycin for *E*. *coli*, isolated from an astronaut aboard Salyut 7, increased from 4 μg/ml to >16 μg/ml, compared to the ground control, while that of *S. aureus* increased two fold for oxacillin, chloramphenicol, and erythromycin^6^. Increased antibiotic resistance has also been noted on Discovery shuttle and Mir space station flight missions^7^. The mode of action for this resistance is unclear, but it could be due to increased mutations in bacterial genes. A study comparing *S. epidermidis* grown on the ISS vs Earth, for 122 hours, showed a 24-fold higher frequency in mutations in the *rpoB* gene (beta subunit of RNA polymerase) when grown on the ISS, which rendered the isolates resistant to rifampicin^8^.

In a study comparing anti-microbial resistance in bacteria isolated from the ISS and the Antarctic Research Station Concordia, Schiwon *et al*. found that 75.8% of the ISS isolates were resistant to one or more antibiotics, whereas only 43.6% of the Antarctic isolates were resistant^9^. From the ISS, 86.2% of the isolates had relaxase and/or transfer genes encoded on plasmids which can promote the dissemination of AMR genes from one microorganism to another^9^. Importantly, the same genera and even most of the same species were examined between the two locations. The two major differences between these two confined, hostile and extreme environments are microgravity and radiation, which may contribute to the higher prevalence of antibiotic resistant microorganisms on the ISS. However, unlike the Schiwon *et al.*^9^ study that measured AMR profiles from isolated strains, this study has examined AMR gene profiles directly from DNA, without culturing.

It is well established that only a small proportion of microorganisms in any given environment can be cultured with standard laboratory techniques and the ISS is no different^2^. A recent microbial tracking study performed by our lab showed that less than 30% of intact, potentially viable microorganisms (i.e. samples treated with propidium monoazide (PMA) which prevent amplification of dead cells in molecular analyses) could be cultured, leaving 70% of organisms and their resistomes (i.e. the collection of AMR genes within a genome) unaccounted for (Checinska Sielaff *et al*., manuscript in preparation). In a recent study, propidium iodide ions were shown to move across intact cell membranes for *Dinoroseobacter shibae* and *Bacillus subtilis* using BacLight fuorescence staining and hypothesized that high membrane potential facilitated breakthrough of the double-charged propidium iodide and marked viable cells as dead^10^. In contrast, using polymerase chain reaction (PCR) we have shown that PMA was able to intercalate DNA in compromised cells which eventually blocked the amplification^11^.

In this study, we performed a thorough analysis of anti-microbial resistance genes within the ISS environment, using traditional and state-of-the art molecular techniques. Samples from eight ISS locations were obtained from three separate sampling events. The bacteria isolated from the samples were tested for resistance to several antibiotics. Concurrently, DNA from PMA treated samples was subjected to a target amplification of 518 antimicrobial genes, designed to match the content of the Antimicrobial Resistance Determinant Microarray (ARDM)^12^. Samples were treated with PMA before DNA extraction so that our focus would be on intact, potentially viable cells and not dead ones^11^ as live cells pose a greater risk to astronaut health. In addition, metagenome data generated from the DNA of the same PMA treated samples were mined for the presence of AMR and compared. This is the first report that presents the identification of the most common AMR genes on the ISS without culturing and correlates the results with phenotypic susceptibility and WGS data of cultured isolates. The study of the ISS resistome is crucial for the development of better surveillance and mitigation procedures on future long-duration spaceflight.

## Results

**Figure 1:**
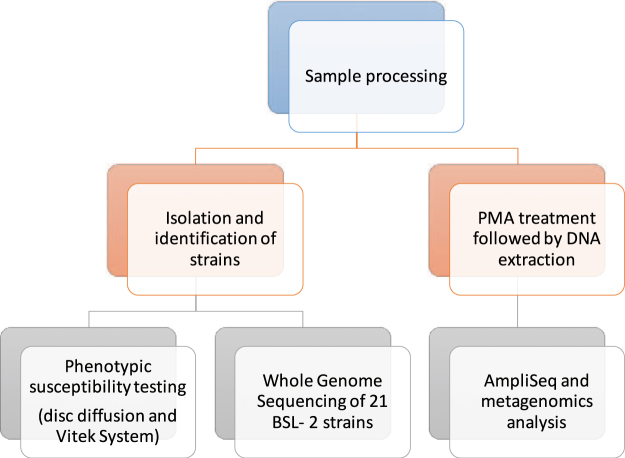
Schematic of analyses performed on 24 samples collected from various locations across the ISS over 3 flight missions.

Twenty-four samples were collected during three sampling events, over a span of a year, from eight locations onboard the ISS (Table 1). Samples were both (i) cultured, to obtain isolates for phenotypic antibiotic susceptibility testing and WGS and (ii) subjected to DNA extraction, after propidium monoazide (PMA) treatment, to allow for Ion AmpliSeq™ and metagenomics analyses. PMA is a dye that intercalates with nucleic acid from dead cells and free DNA, allowing one to assess only intact bacteria within a sample^11^. A summary of the analysis work flow is presented in Fig. 1.

**Table 1:** Description of ISS locations and associated metadata, from which surface swabs were collected.

Location number	Location description	ISS module	
1	Port panel next to cupola	Node 3	
2	Waste and hygiene compartment	Node 3 “F4”	
3	Advanced resistive exercise device (ARED) foot platform	Node 3	
4	Dining table	Node 1	
5	Overhead 4	Node 1	
6	Permanent multipurpose module (PMM) Port 1	PMM	
7	Lab 3 overhead	LAB	
8	Port crew quarters, bump out exterior aft wall	Node 2	
**Environmental parameters**	**Flight 1 (F1)**	**Flight 2 (F2)**	**Flight3 (F3)**
Sampling date	March 4^th^ 2015	May 15^th^ 2015	May 6^th^ 2016
Vehicle (Ascent/Descent)	SpX-5/TMA-14A	SpX-6/SpX-6	SpX-8/SpX-8
Crewmember who performed sampling	T. Virts	T. Virts	J. Williams

### Phenotypic susceptibility tests of cultured organisms

Fifty-seven Biosafety Level-2 (BSL-2) microorganisms, isolated from the ISS, were tested for their resistance against the following 9 antibiotics using the disc diffusion method: cefazolin, cefoxitin, ciprofloxacin, erythromycin, gentamycin, oxacillin, penicillin, rifampin and tobramycin. Table S1 shows the measured zone of inhibition along with the percentage of resistant, intermediate resistant and susceptible strains to each antibiotic. Out of the total 57 strains tested, 92% were resistant to penicillin while only 14% were resistant to gentamicin and ciprofloxacin. For the remaining 6 antibiotics the resistance percentages were as follows: oxacillin (68%), rifampin (66%), erythromycin (64%), cefoxitin (49%), cefazolin (29%) and tobramycin (19%). *Enterobacter bugandensis*, a novel species isolated from the ISS (manuscript in preparation), was resistant to the most antibiotics: all 6 *E. bugandensis* isolates tested were resistant against cefazolin, cefoxitin, erythromycin, oxacillin, penicillin and rifampin, while for ciprofloxacin and gentamycin, strains were either resistant or intermediate resistant. For tobramycin some strains were resistant, some intermediate resistant and some susceptible. *S. haemolyticus* on the other hand, was not resistant to any of the antibiotics tested. Due to the serious health concerns of methicillin resistant *S. aureus* (MRSA), which are resistant to a variety of antibiotics, all 12 *S*. *aureus* isolates obtained on the ISS were further tested with the Vitek system, which included a more comprehensive panel of antibiotics. Antibiotics that were tested with both the disc diffusion assay and Vitek showed the same results (with the exception of three isolates which were susceptible with Vitek but resistant with the disc assay) and the additional antibiotics tested with Vitek showed all strains to be susceptible, except four that were resistant to clindamycin (Table S1). In addition, assessment of methicillin resistance (i.e. MRSA) was performed using the HardyCHROM MRSA biplates and all 12 *S*. *aureus* strains tested were shown to not be MRSA.

### Examining bacterial genomes for antibiotic resistance genes

**Figure 2:**
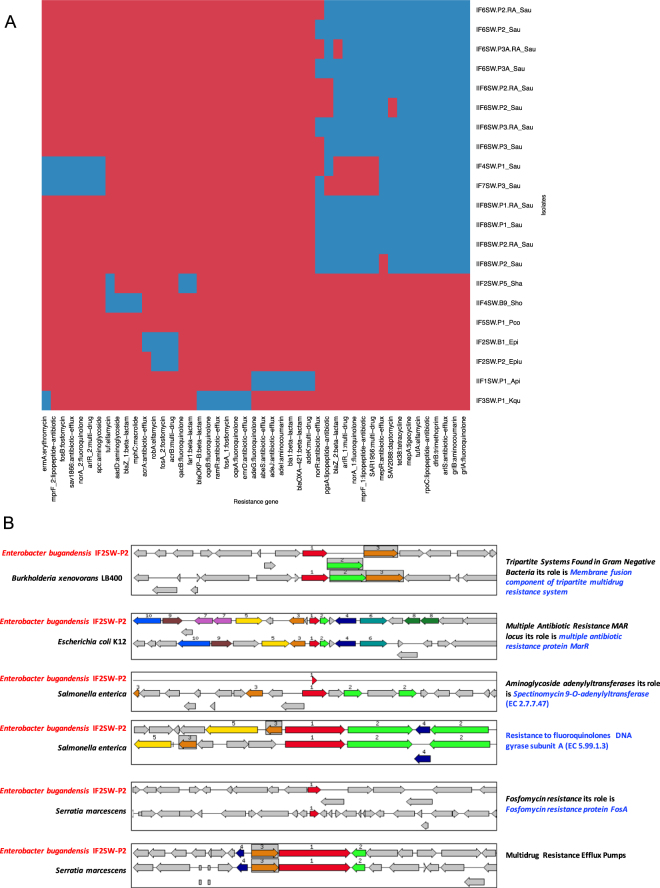
Whole genome sequencing analysis of 21 BSL-2 strains isolated from the ISS. (**A**) The heatmap shows the gene presence (blue) and absence (red) profile of anti-microbial resistance (AMR) genes in whole genome sequenced isolates. Rows represent isolates and columns report the AMR genes found in that specific isolate. Genes with at least 90% nucleotide identity and 90% of the query gene aligned were considered to be positive matches. Resistance genes include the gene name and function class. Where multiple distinct genes of the same type were identified a numeric integer (e.g. 1, 2) is used to denote the difference. Sau: *Staphylococcus aureus*, Sha: *Staphylococcus haemolyticus*, Sho: *Staphylococcus hominis*, Pco: *Pantoea conspicua*, Epi: *Enterobacter bugandensis*, Api: *Acinetobacter pitti*, and Kqu: *Klebsiella quasipneumoniae*. Naming nomenclature: IF and IIF refers to isolates cultured from Flight 1 and Flight II samples respectively. The number after IF or IIF refers to the location sampled. For example IF6SW.P2.RA_Sau refers to *S.aureus* cultured from Flight 1, location 6 sample. (**B**) Antibiotic resistance genes in *Enterobacter bugandensis* IF2SW-P2, isolated from location 2 (waste and hygiene compartment), during Flight 1. Six specific antibiotic resistance gene clusters were shown to be present in four pathogens but absent from *Enterobacter* species isolated on Earth. Annotations were done using the NCBI Prokaryotic Genome Annotation Pipeline and visualized with SEED viewer^50^. Chromosomal region window (16000 bp) is centered on the focus gene, which is red and numbered one. Set of gene with high similarity in sequence are grouped with same color and number. Grey represent genes with similar relative position and functionally coupled.

WGS was performed on 15 of the 57 BSL-2 organisms that were isolated from the ISS. In addition, six *S. aureus* mutant colonies that were present within the clear zone of inhibition after exposure to rifampin, also had their genomes sequenced. The presence or absence of AMR genes in each isolate is displayed in Fig. 2A. It is evident that *S. aureus* genomes show a distinct AMR gene profile compared to other staphylococcal species and other genera. Furthermore, amongst *S. aureus* isolates, the two that were resistant to erythromycin (IF4SW_P1_Sau and IF7SW_P3_Sau), as determined by the disc diffusion assay and Vitek testing, display a distinct AMR gene profile compared to all the others, indicating a possible genetic basis for resistance.

Mutations in the *rpoB* gene have been implicated in rifampin resistance^8^, therefore the *rpoB* gene sequence from the spontaneous rifampin resistant mutants and the sensitive parent strains were compared. With the exception of a base pair change from C to T at position 144, there were no other differences between parent and mutant strains.

Seven antibiotic resistance gene clusters were analyzed in the novel species *E. bugandensis*, isolated from the waste and hygiene compartment on the ISS (manuscript in preparation). These AMR gene clusters were associated with known pathogens and were not found in *Enterobacter* species isolated on Earth (Fig. 2B). This suggests that horizontal gene transfer (HGT) might have occurred but more research is needed to determine if HGT happened on the ISS.

### Anti-microbial resistance gene profiles on the ISS using Ion AmpliSeq^™^

To survey the AMR gene content of a large collection of samples and independent of culture restrictions, DNA isolated from the 24 ISS samples was analyzed using Ion AmpliSeq™. Ion AmpliSeq™ technology consists of a pool of primer pairs, with each pair designed to amplify a specific gene. In our study, this panel consisted of primer pairs designed against 518 known antibiotic resistance genes from a variety of bacteria^12^. Of the 518 genes that were tested for, a total of 123 were detected, but only those that were over 100 reads from the Ion Torrent sequencer were kept for further analysis. This was a threshold setting to remove potential “false positives” which can arise from non-specific primer binding, mutations during amplification and Ion Torrent sequencing error^13,14^. A comprehensive summary of the 63 detected genes (those with read counts over 100), their annotation numbers in NCBI (GI), the microorganisms they are related to, their function and the resistance they confer are found in Table S2.

**Figure 3:**
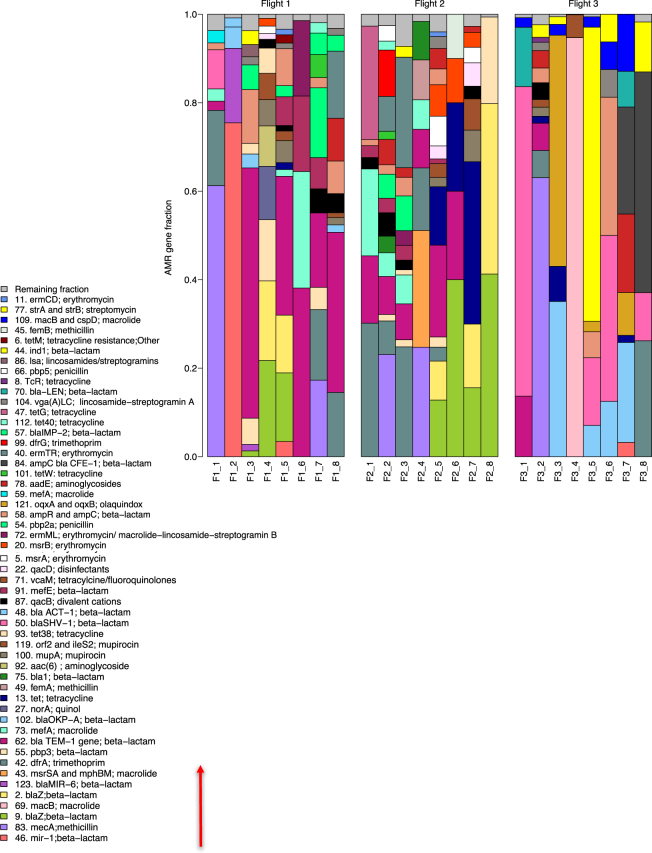
Relative abundance of anti-microbial resistance genes (AMR) aboard the ISS. Bar plot showing the relative abundances of AMR genes in samples collected from different locations aboard the ISS, during three sampling events. F refers to the flight number, followed by the location (i.e. F1_1 is Flight 1 location 1 (see Table 1 for more details)). Each color represents a gene and the height of the colored box represents the proportion of that gene within that sample. The remaining fraction, colored grey, groups genes that were less than 1% abundance in that sample. The legend shows the gene name (i.e. *mecA*) followed by the antibiotic that gene confers resistance to (i.e. methicillin). The number in front of the gene name is the personal identifier that can be matched to the metadata listed in Table S2. The legend is read from bottom to top, as indicated by the red arrow, with the bottom AMR gene on the legend corresponding to the bottom colored box on the bar plot.

**Figure 4:**
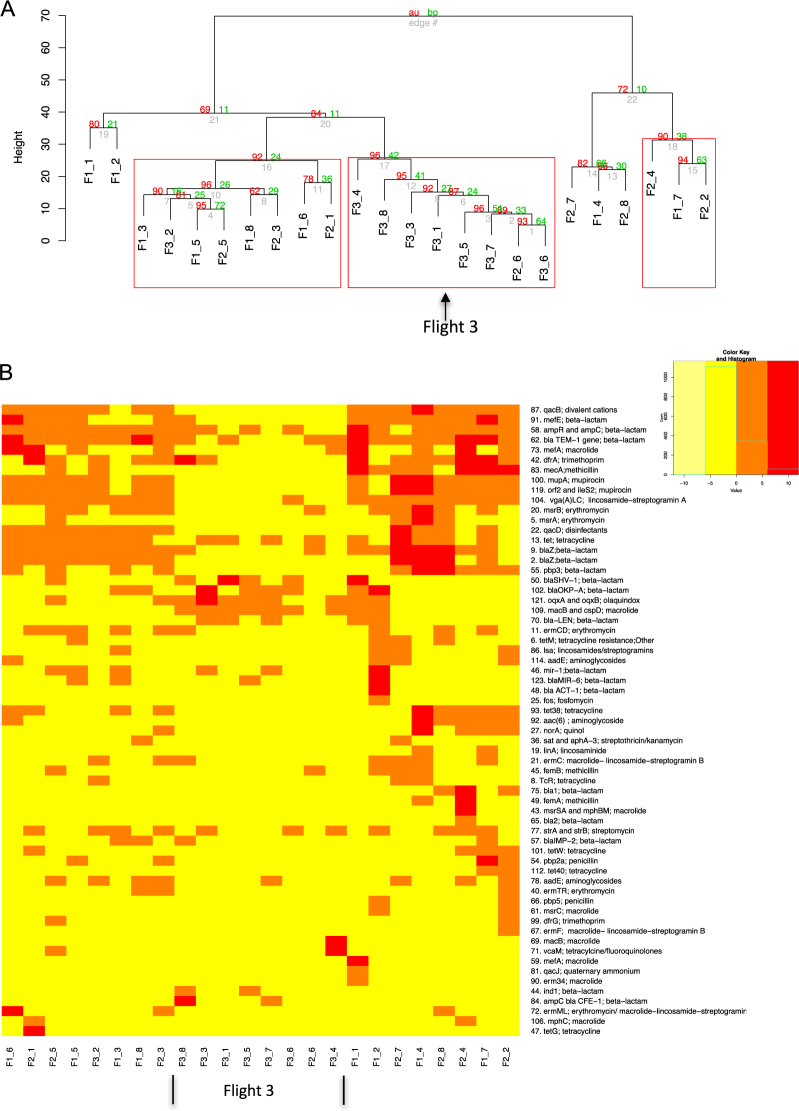
Temporal comparison of AMR gene profiles. (**A**) Hierarchical clustering of Euclidian distances of centered log ration transformed (clr) data, using the Ward clustering method. Red values are **AU (A**pproximately **U**nbiased) *p*-values, and green values are **BP (B**ootstrap **p**robability) values. Clusters with AU p-values larger than 90% are highlighted by rectangles (significance level 0.10), meaning that there is a 90% certainty of these clusters being a distinct group. F”x” refers to the flight number, followed by the location (i.e. F1_1: Flight 1, location 1). (**B**) Heat map of centered log ratio transformed data. Red and orange boxes indicate samples that have genes with relative abundances higher than the geometric mean abundance (represented as 0), which has been calculated from all samples. Simply, it means that these samples have a high abundance overall. Boxes in yellow indicate samples having genes with relative abundances that are lower than the geometric mean abundance (i.e. low abundance overall). The columns show the samples and the rows show the gene, followed by the antibiotic that the gene confers resistance to. The number in front of the gene name is the personal identifier that can be matched to the metadata listed in Table S2 and to the genes shown in Fig. 3.

Figure 3 shows the relative proportion of each detected AMR gene within a sample, with each colored bar representing a different AMR gene. The different colors and color patterns for each location suggest differences in spatial distribution of AMR genes at each location. This variability was confirmed with biplots, which show the distribution of the samples in relation to each other, and the contribution of each variable (i.e. gene) to the overall composition of that sample (Fig. S1). In addition to spatial differences, the dendrogram in Fig. 4A, shows temporal differences as well, with a clear separation of Flight 3 (F3) samples from Flight 1 (F1) and Flight 2 (F2). While there was no overall separation between F1 and F2, most paired samples (i.e. F1_8 vs F2_8) do cluster apart between the two sampling events (i.e. locations 1, 2, 4, 6, 7, 8). The separation between F1/F2 and F3 may be attributed to the low relative abundances of AMR genes present on the ISS during F3. As the heatmap in Fig. 4B shows, F3 samples had a higher number of genes shaded yellow, an indicator of abundances lower than the geometric mean abundance of all the 24 samples combined.

**Figure 5:**
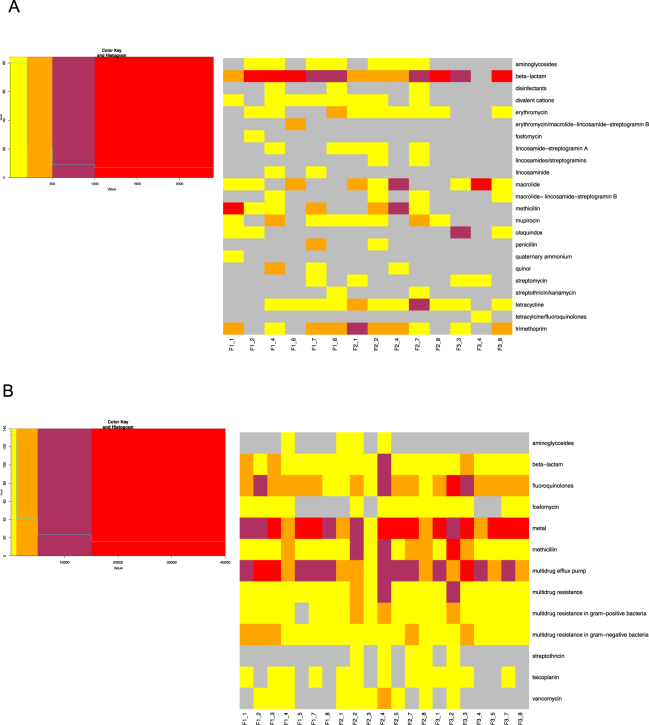
Distribution of antibiotic resistance across samples at the function level from both Ion AmpliSeq™ and metagenomics analysis. (**A**) Heat map showing read counts of AMR genes grouped together based on the class of antibiotic they are resistant to, as detected by AmpliSeq™. Samples were rarefied to 1830 reads, with those lower than 1830 read counts removed during rarefaction. (**B**) Read counts of AMR genes grouped together based on class of antibiotic they are resistant to, as detected by metagenomics. Grey boxes indicate genes that were absent in a particular sample. Red indicates the highest read count and yellow, the lowest read count. Columns represent samples and rows, antibiotic resistance.

At the functional level, the genes for beta-lactam and trimethoprim resistance were abundant and widespread across the ISS (Fig. 5A). However, when a metagenomics approach was used to examine the AMR gene profiles, genes for beta-lactam resistance, while found in every sample, were not as abundant compared to other functional groups and genes for trimethoprim resistance was not detected (Fig. 5B). The metagenomics approach also showed that genes conferring resistance to metals (i.e. zinc, copper etc) and those involved in multi-drug efflux pumps were the most abundant across the ISS, both of which were not detected with Ion AmpliSeq™. Fluoroquinolone resistance was also highly represented across the ISS, while Ion AmpliSeq™ only detected this gene family in only 3 samples. Ion AmpliSeq™ detected 23 antibiotic groups and metagenomics 13, with 6 shared between the two methods: aminoglycosides, beta-lactams, quinols (i.e. fluoroquinolones), fosfomycin, methicillin and streptomycin. At the gene level AmpliSeq™ and metagenomics both detected *tetR*, *macB*, *fosA*, and *femB*. As the two methods use a different set of reference genes and the raw data is processed differently, the fact that there are differences is not surprising, but it does highlight the usefulness of using a variety of analytical approaches.

Multiple AMR genes can be transferred on a single plasmid, giving rise to multi-drug resistance in the recipient. We were therefore interested in assessing whether any genes were associated with each other and thus possibly found together. A correlation analysis showed statistically significant negative and positive correlations between the genetic determinants of AMR (see Table 2 for Spearman rho and *P* values).

**Table 2:** Summary of correlation analysis between AMR gene function. Positive spearman rho values indicate positive correlations and negative values indicate negative correlations.

Genes responsible for the following resistance	Genes responsible for the following resistance	p-value	Spearman rho value
mupirocin	lincosamide-streptogramin A	0.005	0.7
mupirocin	Divalent cations	0.03	0.6
fosfomycin	olaquindox	0.04	0.6
olaquindox	Divalent cations	0.03	−0.6
olaquindox	mupirocin	0.01	−0.6
macrolide	lincosamide-streptogramin A	0.004	−0.7

## Discussion

We have provided a comprehensive summary of the AMR genes present across the ISS over a span of a year using phenotypic assays, WGS, Ion AmpliSeq™ and metagenomics. The WGS of BSL-2 isolates showed the collective presence of 51 genes conferring resistance to 18 antibiotics. In most cases, the genotype of the sequenced strains could predict the phenotype, such as the presence of *ermA* and *blaZ* in *S. aureus*, which protects against erythromycin and penicillin respectively^15^ and the presence of *oqxA* and *oqxB* in *K. quasipneumoniae*, which protects against ciprofloxacin^16^, all of which were resistant to their respective antibiotics in the disc diffusion assay. In other cases, the AMR profiles were not as straightforward to interpret. For example, one *S. aureus* isolate, (IF4SW.P1_Sau) lacking the *blaZ* gene was still resistant to penicillin. In another case, the presence of *norA* in multiple *S. aureus* isolates did not protect against ciprofloxacin, even though transfer of this gene to susceptible strains has been proven to be enough to confer resistance^17,18^. The lack of consensus between genotype and phenotype in this case could be due to inadequate gene expression, which has been shown in numerous studies to be a barrier against resistance^16^. Moreover, this study was focused on the detection of genomic sequences of known AMR genes so any mutations and variations in gene expression of AMR genes were not analyzed in this study.

*E. bugandensis* was the most antibiotic resistant species tested with our phenotypic assay, with all six isolates being resistant to all antibiotics tested (with the exception of two that were susceptible to tobramycin). *Enterobacter* species, a member of the coliform group of bacteria, have been reported to be pathogenic and cause opportunistic infections in immunocompromised individuals and are commonly associated with urinary and respiratory tract infections^19^. Of note, approximately 6% of the isolates cultured from the ISS were identified as *Enterobacter*. Carbapenems, a class of beta-lactams, are the most effective treatment against *Enterobacter* infections as they remain stable against extended spectrum beta lactamases, unlike penicillin and oxacillin. Ertapanem is the only carbapenem included in the medical kit onboard the ISS, however this may need to be re-evaluated, as numerous clinical isolates of *Enterobacter* species have shown resistance to ertapanem, though not other carbapenems, such as imipenem and meropenem^20,21^. There is the possibility that our ISS *Enterobacter* isolates, like the clinical isolates, are resistant to ertapanem, and potentially other carbapenems, as the IMP-2 gene, a metallo beta-lactamase that is able to inhibit the action of this class of antibiotic, was detected in our samples^22^. Further evaluation of this resistance profile and any other multi-drug resistant microorganisms, onboard the ISS, should be made a priority, in order to assess risk of infection and to ensure that proper strategies are in place to combat such infections.

Ion AmpliSeq™ revealed a collective of 65 AMR genes onboard the ISS, representing resistance to 23 different antibiotics. Nine of those detected genes (*qacB*, *blaZ, bla- OKP*, *oqxA, oqxB*, *fos*, *tet38*, *norA* and *bla1*) were also identified in the WGS analysis of our selected cultured isolates. One of the detected genes was *mecA*, a biomarker for methicillin resistant strains of *S. aureus*, commonly referred to as MRSA. MRSA are strains of *Staphylococcus* that have become resistant to methicillin and other beta-lactams (i.e. oxacillin, penicillin) used to treat ordinary *Staphylococcus* infections can cause serious complications (skin infections, sepsis and pneumoniae) in immune-compromised individuals. Even though *mecA* was detected in numerous samples across the ISS via AmpliSeq^TM^, none of the 12 *S*. *aureus* isolates tested with Vitek were MRSA positive nor was *mecA* detected in their genomes. While *mecA* is commonly associated with *S. aureus*, it has also been detected in other *Staphylococcus* species^23,24^ which may have been the source of the detected *mecA* during this study. Despite the fact that none of the *S. aureus* isolates were MRSA at the time of collection, there is the possibility that they acquire *mecA* from other staphylococci. Indeed, the results of a hospital in the Netherlands, showed that *mecA* gene transfer occurred in a patient between *mecA*^+^ *S. epidermidis* and a *mecA*^-^ strain of *S. aureus*^25^. This is not surprising as there is mounting evidence in favor of the “resistance gene reservoir” where commensal organisms in the body act as reservoirs for AMR genes that can be transferred to resident or transient bacteria, including pathogens^26,27^. Further studies assessing the proportion and types of mobile genetic elements within the ISS metagenome could shed some light on how readily AMR (and possibly virulence genes) may be acquired and transferred by bacteria on the ISS and if space conditions promote the transfer of AMR genes between different species and even different genera more readily then when these organisms are grown together on Earth.

A positive correlation was observed between oliquadox (*oqxA* and *oqxB*) and fosfomycin (*fosA*) resistance on the ISS, which conforms with ground studies showing the co-transfer of *oqxA*/*oqxB* and *fosA* genes from single plasmids^28,29^. A positive correlation was also observed between mupirocin and lincosamide/streptograminA resistance genes on the ISS. While studies have documented the co-transfer of β-lactam, quinolone, aminoglycoside, amphenicol and fosfomycin resistance genes to transconjugants^28^, this is the first study, to our knowledge, that has observed an association between muprirocin and lincosamide/streptogtanimA resistance genes.

It has been documented that metal exposure and the resulting resistance towards those metals leads to co-selection of unrelated antibiotic resistance^30,31^, most likely a consequence of metal resistance genes and AMR genes present on the same plasmid and/or transposon^32^. Indeed, bacterioplankton isolated from metal contaminated Ash basins were significantly more tolerant to a variety of antibiotics, which were not found in these basins, compared to bacterioplankton collected from basins void of metals^33,34^. Similar phenomena have been reported from metal contaminated freshwater^34^ and soil samples^35^. Our metagenomics data showed that the highest resistance genes across all time points and all locations were in fact metal resistance genes. It is unknown whether the material of the ISS surfaces that these organisms were collected from promotes metal resistance dissemination amongst the microbiota but if that is the case, this metal resistance may play a role in the spread and abundance of AMR genes on the ISS.

To summarize, this is the first study to utilize an array of molecular methods to characterize the resistome on the ISS unveiling the presence of numerous genes conferring resistance to 28 different antimicrobial agents. The results from this study highlight the importance of using a variety of analytical methods to get a comprehensive picture of the resistome on the ISS and to utilize this information to develop concrete antibiotic resistance mitigation strategies. Some of these strategies could include the development of bioinspired alternative drugs, cleaning procedures for the eradication of specific organisms, bacterial specific microbial monitoring and evaluation of actual doses of antibiotics to be given in space. This will help maintain crew health, especially on long duration flight missions, when return to Earth for treatment is not an option.

## Methods

### Sample kit preparation and sample collection

Sampling wipes were assembled at the Jet Propulsion Laboratory (JPL) following the procedure outlined by^36^. Briefly, each polyester wipe (9″ × 9″) (ITW Texwipe, Mahwah, NJ) was folded two times and soaked in 15 mL of sterile molecular grade water (Sigma-Aldrich, St. Louis, MO) for 30 minutes followed by transfer to a sterile zip lock bag. At the Ames Research Center (ARC, Moffett Field, CA), the wipes were packed along with other items, such as sterile gloves, as part of a kit sent to the ISS. Each sampling kit was then sent to the ISS on the Space-X cargo, and returned to the Earth on Soyuz TM-14 or Dragon capsule (Table 1). The kits were delivered to JPL immediately upon return to Earth.

During each sampling session on the ISS, only one astronaut collected samples from eight different locations. The description of each location is depicted in Table 1. Each wipe was used to collect a sample of one square meter. The control wipe (environmental control) was only taken out from the Zip lock bag, unfolded and packed back to the zip lock. The samples were stored at 4 °C until the return trip to Earth and subsequent processing.

### Sample processing

The sample processing took place in a 10 K class cleanroom at JPL immediately upon return to Earth. Each wipe was aseptically taken out from the zip lock bag and transferred to a 500-mL bottle containing 200 mL of sterile PBS. The bottle with the wipe was shaken for two minutes followed by concentration with a Concentrating Pipette (Innova Prep, Drexel, MO) using 0.45 µm Hollow Fiber Polysulfone tips and PBS elution fluid. The environmental control and each sample were concentrated to 5 mL.

#### PMA treatment and DNA extraction

A 1.5-mL aliquot was treated with PMA to allow for amplification of DNA from only intact cells^11^. 18.25 µL of 25 µM PMA in water was added to each sample followed by 5-minute incubation at room temperature in the dark, followed by a 15-minute exposure to the activation system (PMA LED device, Biotium, Hayward, CA). 750 µl was then transferred to bead beating tubes containing Lysing Matrix E (MP Biomedicals, Santa Ana, CA) and subjected to bead beating for 60 s using the vortex sample holder (MO Bio, Carlsbad, CA). The bead-beaten half and the unprocessed half were combined, followed by DNA extraction with the Maxwell 16 automated system using the Maxwell 16 Tissue LEV Total RNA purification kit in accordance with the manufacturer’s instructions (Promega, Madison, WI). Resulting DNA samples in water (50 μL each) were stored at −20 °C until downstream analysis.

#### Isolation and identification of bacteria from samples

The concentrated samples were diluted in PBS (up to 10^−6^ of each original sample) and 100 µL (in duplicate), were plated on R2A agar for environmental bacteria (incubated at room temperature for 7 days), and blood agar (BA) for human commensals (incubated at 35 °C for two days). On average, five isolates of distinct morphologies were picked up for each location from each type of media. The isolates were archived in semi solid R2A slants (dilution 1:10) and stored at room temperature. For identification, each bacterial isolate was revived on R2A media. Once a culture was confirmed to be pure, DNA extraction was performed by colony PCR, UltraClean DNA kit (MO Bio, Carlsbad, CA) or Maxwell Automated System (Promega, Madison, WI). The 1.5 kb 16S rRNA gene was used to identify the bacterial isolates using the following primers: 27 F (5′-AGAGTTTGATCCTGGCTCAG-3′) and 1492 R (5′-GGTTACCTTGTTACGACTT-3′). The PCR conditions were as follows: denaturation at 95 °C for 5 min, followed by 35 cycles consisting of denaturation at 95 °C for 50 s, annealing at 55 °C for 50 s, and extension at 72 °C for 1 min 30 s and finalized by extension at 72 °C for 10 min. When 16S rRNA identification was ambiguous (*B. cereus* group isolates), the primers for *gyrB* (UP-1: 5′-GAA GTC ATC ATG ACC GTT CTG CAY GCN GGN GGN AAR TTY GA-3′ and UP-2R: 5′-AGC AGG ATA CGG ATG TGC GAG CCR TCN ACR TCN GCR TCN GTC AT-3′) were used and a 1.2-kb fragment of gyrase B was amplified as described previously^37,38^. The sequences were assembled using SeqMan Pro from DNAStar Lasergene Package (DNASTAR Inc., Madison, WI). The bacterial sequences were searched against EzTaxon-e database^39^ and identified based on the closest similarity to 16S rRNA sequences of bacterial type strains.

### Whole Genome Sequencing of Opportunistic Strains

Genomic DNA (gDNA) extracted from 21 BSL-2 bacterial strains were sent to Lawrence Livermore National Laboratory for WGS using the Illumina NextSeq. 500 sequencing system (San Diego, CA). The concentration of the gDNA was determined using a Qubit fluorimeter (ThermoFisher Scientific, Waltham, MA). The sequencing library preparation was performed on the gDNA samples using the TruSeq DNA PCR-Free LT kit (Illumina, San Diego, CA) according to the manufacturer’s protocol. Briefly, the concentration of each gDNA was normalized to 40 ng/µl and 55 µl of each sample was fragmented on a Covaris M220 ultrasonicator (Covaris, Woburn, MA) using settings to generate 550 base pair inserts. The Illumina TruSeq protocol was further followed to repair the fragmented DNA ends, adenylate the 3’ ends of the fragments, and finally ligate Illumina sequencing adaptors to the fragmented DNAs.

The libraries were validated using the Library Quantification Kit for Illumina platforms (KAPA, Wilmington, MA) following manufacturer’s protocols. Following the quantification, each library was normalized, pooled, and denatured according to Illumina protocols. Briefly, each library was normalized to 0.5 nM and 5 µl of each normalized sample was pooled together. The 40-µL pooled sample was denatured by combining with 40 µL of 0.2N NaOH. The denatured, pooled sample was further diluted to a concentration of 1.8 pM where it was mixed with denatured PhiX control DNA (Illumina, San Diego, CA) at a concentration of 1%. This sample was loaded on to the NextSeq Sequencer using the NextSeq High Output v2 300 cycle sequencing kit (Illumina, San Diego, CA). The NextSeq was set up to run a paired end, 150 nucleotide (2 × 150) run with single indexing.

#### Data assembly and gene annotation

Draft assemblies of the bacterial isolates were generated as described in^40^. Starting with the draft assemblies as input, genes were predicted for each genome using Glimmer v. 3.02^41^ using the g3-from-scratch.csh script with default settings to generate genome specific gene models. Each gene was searched against a collection of curated genes taken from the comprehensive antibiotic resistance database (CARD)^42^, ResFinder^43^ and ARG-ANNOT^44^. Each gene was searched against the curated gene database using the top hit from a blastn search (NCBI BLAST v2.2.27+) with word_size 8, num_threads 20, outfmt 7, max_target_seqs. 1, and all other options set to default. A table was generated recording individual genes that matched to an annotated resistance gene for each organism with at least 90% identity between the query and reference gene over at least 90% of the length of reference gene. Gene labels and gene function were manually assigned based on annotation assignments given in the included curated gene databases.

### Phenotypic antibiotic resistance testing

The isolates were streaked from glycerol stocks onto R2A plates. A single colony was inoculated into 5-mL Tryptic Soy Broth (TSB) and grown overnight at 30 °C. Aliquots of 100 µL were plated on TSA. Agar diffusion discs (BD BBL^TM^ Sensi-Disc^TM^, Franklin Lakes, NJ) were placed aseptically on a plate and the strains were incubated at 37 °C for 24 hours. The tested antibiotics included: 30-µg cefazolin (CZ-30); 30-µg cefoxitin (FOX-30), 5-µg ciprofloxacin (CIP-5), 15 µg erythromycin (E-15), 10-µg gentamicin (GM-10), 1-µg oxacillin (OX), 10-µg penicillin (P-10), 5-µg rifampin (RA-5), and 10-µg tobramycin (NN-10). The diameter of inhibition zones was measured for each antibiotic disk and recorded in millimeters. The resistance results were compared with the zone diameter interpretive charts provided by the manufacturer. When the spontaneous mutants were present in response to some antibiotics, they were isolated, subcultured and tested for the specific antibiotic resistance.

Vitek®2 Compact AST-GP67 cards (BioMerieux, Inc., Hazelwood, MO) were selected to test the *S. aureus* isolates. The AST-GP67 cards are designed for testing gram-positive cocci. The tested antibiotics were ampicillin, benzylpenicillin, beta-lactamase, cefoxitin, ciprofloxacin, clindamycin, erythromycin, gentamicin, levofloxacin, linezolid, moxifloxacin, nitrofurantoin oxacillin, quinupristin/dalfopristin, rifampicin, streptomycin, tetracycline, tigecycline, trimethoprim/sulfamethoxazole and vancomycin. The cards were initially analyzed for expected outcomes by running the manufacturers recommended quality control organisms *S. aureus* ATCC 29213, ATCC BAA-1026, ATCC BAA-976, and ATCC BAA-977, and the results were as predicted (data not shown). Liquid aliquots of the organisms were shipped overnight from JPL to AlloSource® (Centennial, CO) and refrigerated upon arrival. The aliquot of 0.1 mL subcultures of each microorganism was spread onto Trypticase Soy Agar (TSA) plates (BD BBL^TM^) and incubated overnight at 30–35°C. Using a sterile cotton swab, a pure colony of each organism was suspended in 3.0 mL sterile 0.9% saline at 0.55 McFarland using a DensiCheck® densitometer (BioMerieux, Inc.). A second dilution was prepared by aliquoting 0.28 mL of the initial inoculant into a fresh 3.0 mL sterile 0.9% saline solution. The swab was inoculated onto a TSA plate to check for inoculant purity. Both concentrations of each organism were loaded into separate AST-GP67 cards and analyzed by the Vitek®2 Compact (BioMerieux, Inc.) applying the CLSI+ Natural Resistance susceptibility standards per the manufacturers recommendations. Reports were verified by AlloSource® technicians. Confirmation of methicillin sensitivity (MRSA) was performed using HardyCHROM MRSA biplates (Hardy Diagnostics, Santa Maria, CA). Each isolate was streaked on both sides of the bi-plate and incubated at 30–35 °C overnight. Isolate sensitivity was interpreted using the manufacturers recommendation

### Ion AmpliSeq^™^

#### Library preparation and targeted Sequencing

Ion AmpliSeq™ libraries (Life Technologies) were constructed with the Ion AmpliSeq™ library protocol as previously described^45^. The PCR conditions were as follows: 2X primer mix, 6 µL of sample and 16 PCR cycles consisting of a 99 °C for 15 sec; 60 °C for 4 min. Both primer pools were utilized to create two unique Ion AmpliSeq™ libraries for each metagenome sample. All libraries were quality checked using the Agilent BioAnalyzer and quantitated using the Ion Library Quantitation Kit. Template was diluted to target 10–30% enriched beads and clonally amplified using Ion PGM OT2 400 kit. Enriched beads were sequenced using the Ion PGM 400 Sequencing kit on an Ion 318™ chip at default instrument parameters. Libraries were sequenced in multiplex.

#### *Ion AmpliSeq*™ *panel design*

An Ion AmpliSeq™ custom panel (AmpliSeq MDR v.1, Thermo Scientific, South San Francisco, CA, USA) was designed to match the content of the Antimicrobial Resistance Determinant Microarray (ARDM)^12^. Primer design was performed by Life Technologies using the Ion AmpliSeq™ strategy and the Ion AmpliSeq™ software. The Ion AmpliSeq MDR v.1 consists of two primer pools that are capable of generating a total of 1,358 amplicons targeting 518 known, non-SNP mediated resistance determinants. The assay was designed to generate two unique amplicons per target for 404 resistance genes. For the remaining 114 genes corresponding to *bla* (beta-lactam) and *qnr* (quinolone) resistance determinants, the assay was designed to generate a sufficient number of overlapping amplicons to span the entire gene. Amplicon sequences are available upon request. Primer information is not supplied by Life Technologies.

#### Amplicon sequence read quality control

Sequence read files were imported into CLC Genomics Workbench v 8.0 (CLC Inc, Aarhus, Denmark). Reads were trimmed at default parameters based on quality score (≤0.05), ambiguous nucleotides (≤2) and filtered on read length (≥75 bp). Trimmed reads were saved as a separate file and used in all downstream bioinformatics analyses.

#### Reference mapping

Sequence read mapping was performed using CLC Genomics Workbench v8.0 CLC Reference Mapper was run with default settings (Insertion cost = 3, Deletion cost = 3, Mismatch cost = 2, Length fraction = 0.5 and similarity fraction = 0.8). Mapping of all sequence reads was performed using a multi-fasta file that contained the PCR amplicons that comprise the ARDM array.

The raw sequence reads have been deposited into the NCBI SRA database under accession #SRP082440.

#### Bioinformatics analysis

Dendrogram, heatmaps, barplot and biplots were all generated in R (http://www.R-project.org/). Relative abundances displayed in the bar plot were generated by taking the read count of a gene in a sample and dividing by the total read count of that sample. Data was centered log ratio (clr) transformed^46^ using the “compositions” package in R. The dendrogram was created with the “pvclust” package, which assesses the uncertainty in hierarchical cluster analysis. The heatmap was generated with the “gplot” package. Counts rarefied to 1830 reads were used in the heatmap and were generated in QIIME using the single_rarefaction.py script. Correlation analyses between the microbiome and anti-microbial resistance (AMR) genes were performed as per^47^. Briefly, the Spearman’s rank correlation between the relative abundances of each genus (clr transformed) in 128 inferred technical replicates and clr transformed AMR relative abundances were calculated with the aldex.corr function from the ALDEx2 package in R. Spearman’s rho values were converted to P-values and corrected by the Benjamini-Hochberg method. Correlations between AMR genes were performed as above, with the AMR dataset substituted for the microbiome dataset in the script.

### Metagenome analysis

Extracted DNA was sent to Second Genome (South San Francisco, CA) for sequencing. All samples were quantified via the Qubit® Quant-iT dsDNA High Sensitivity Kit (Invitrogen, Life Technologies, Grand Island, NY) to ensure that they met minimum concentration and mass of DNA. Samples were prepared for sequencing with the Illumina Nextera kit and quantified with Quant-iT dsDNA High Sensitivity assays. Libraries were pooled and run with 100 bp paired-end sequencing protocols on the Illumina HiSeq. 2500 platform. The NGS QC Toolkit v 2.3 was used to filter the raw data for high-quality (HQ) vector- and adaptor-free reads for genome assembly (cutoff read length for HW, 80%; cutoff quality score, 20)^48^. Filtered DNA sequences were mapped against a reference database of all proteins within the KEGG databases using MEGAN6^49^. Reads over 100 for each gene, that were predicted for antimicrobial resistance were clustered together and visualization was done using MEGAN. A total of 8 million reads were generated for each sample. The heatmap used to visualize the data was generated in R using the gplot function.

### Availability of sequence data

The data presented in this manuscript are available in the NCBI Sequence Read Archive under accession number: SRP082440. The raw metagenomics sequences can be downloaded from the following GeneLab website (https://genelab-data.ndc.nasa.gov/genelab/accession/GLDS-66/).

### Disclaimer

This document was prepared as an account of work sponsored by an agency of the United States government. Neither the United States government nor Lawrence Livermore National Security, LLC, nor any of their employees makes any warranty, expressed or implied, or assumes any legal liability or responsibility for the accuracy, completeness, or usefulness of any information, apparatus, product, or process disclosed, or represents that its use would not infringe privately owned rights. Reference herein to any specific commercial product, process, or service by trade name, trademark, manufacturer, or otherwise does not necessarily constitute or imply its endorsement, recommendation, or favoring by the United States government or Lawrence Livermore National Security, LLC. The views and opinions of authors expressed herein do not necessarily state or reflect those of the United States government or Lawrence Livermore National Security, LLC, and shall not be used for advertising or product endorsement purposes.

## Electronic supplementary material

Supplementary File

Table S1

Table S2
