# Supplementary material for: Detection of antimicrobial resistance genes associated with the International Space Station environmental surfaces
Source: Sci Rep. 2018 Jan 16;8:814. doi: 10.1038/s41598-017-18506-4 (PMC5770469; doi:10.1038/s41598-017-18506-4)
Supplement: Supplementary file 1 — Supplementary File [file 41598_2017_18506_MOESM1_ESM.pdf]

Detection of antimicrobial resistance genes associated with the International Space Station environmental surfaces

C. Urbaniak[1], A. Checinska Sielaff[1#], K.G. Frey[2], J.E. Allen[3], N. Singh[1], C. Jaing[3], K. Wheeler[4], K. Venkateswaran[1*]
[1]Jet Propulsion Laboratory, California Institute of Technology, Pasadena, CA
[2]Naval Medical Research Center-Frederick, Frederick, MD
[3]Lawrence Livermore National Laboratory, Livermore, CA
[4]Allosource, Centennial, CO

# current affiliation: Department of Ecology, Evolution, and Organismal Biology, Iowa State University, Ames, IA

*Corresponding author
California Institute of Technology, Jet Propulsion Laboratory
Biotechnology and Planetary Protection Group; M/S 89-2
4800 Oak Grove Dr., Pasadena, CA 91109
Tel: (818) 393-1481; Fax: (818) 3934176
E-mail: kjvenkat@jpl.nasa.gov

Keywords: resistome, antibiotic resistance, International Space Station, AmpliSeq™, metagenomics

Fig S1A

Flight 1

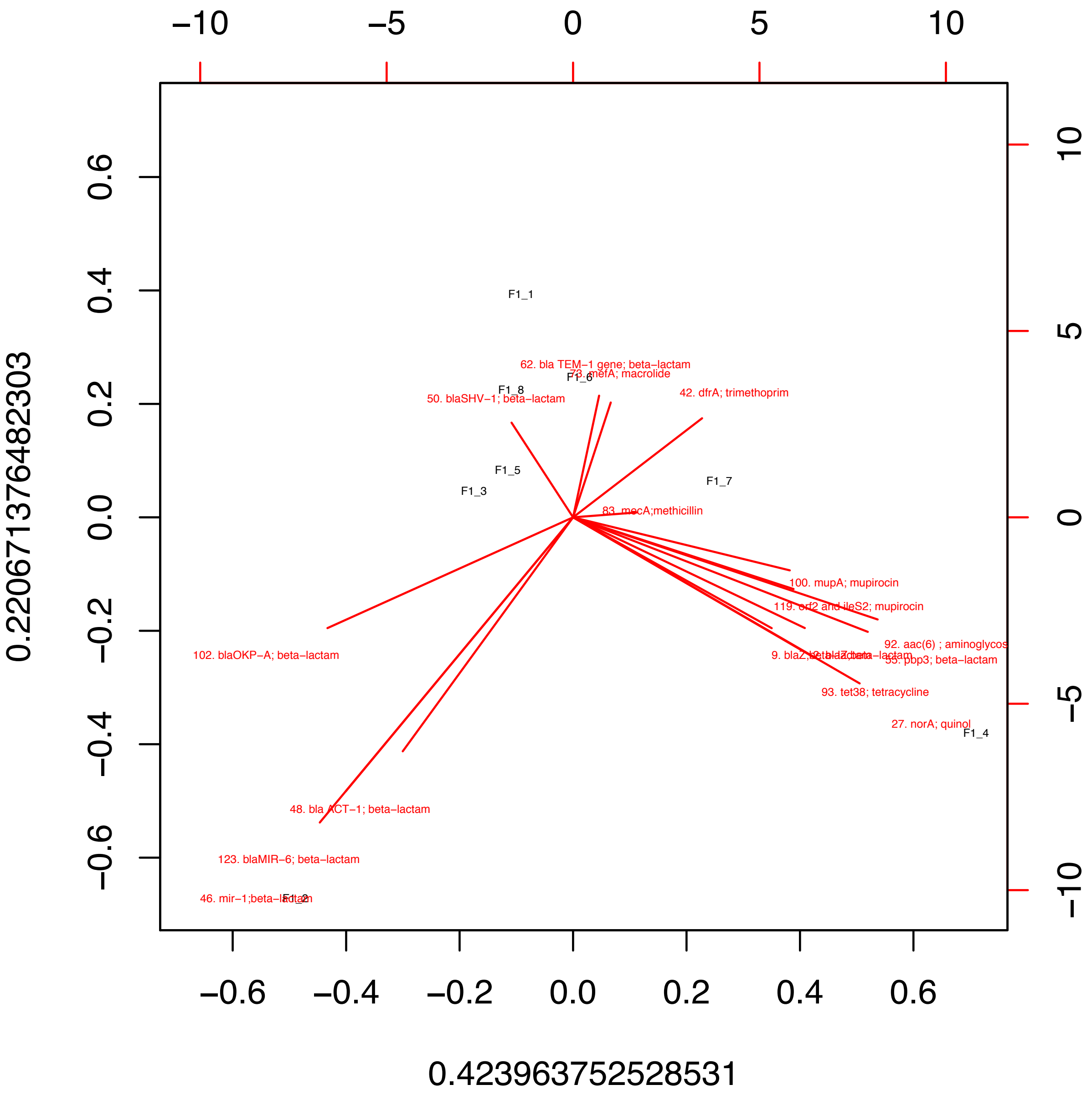

Fig S1B

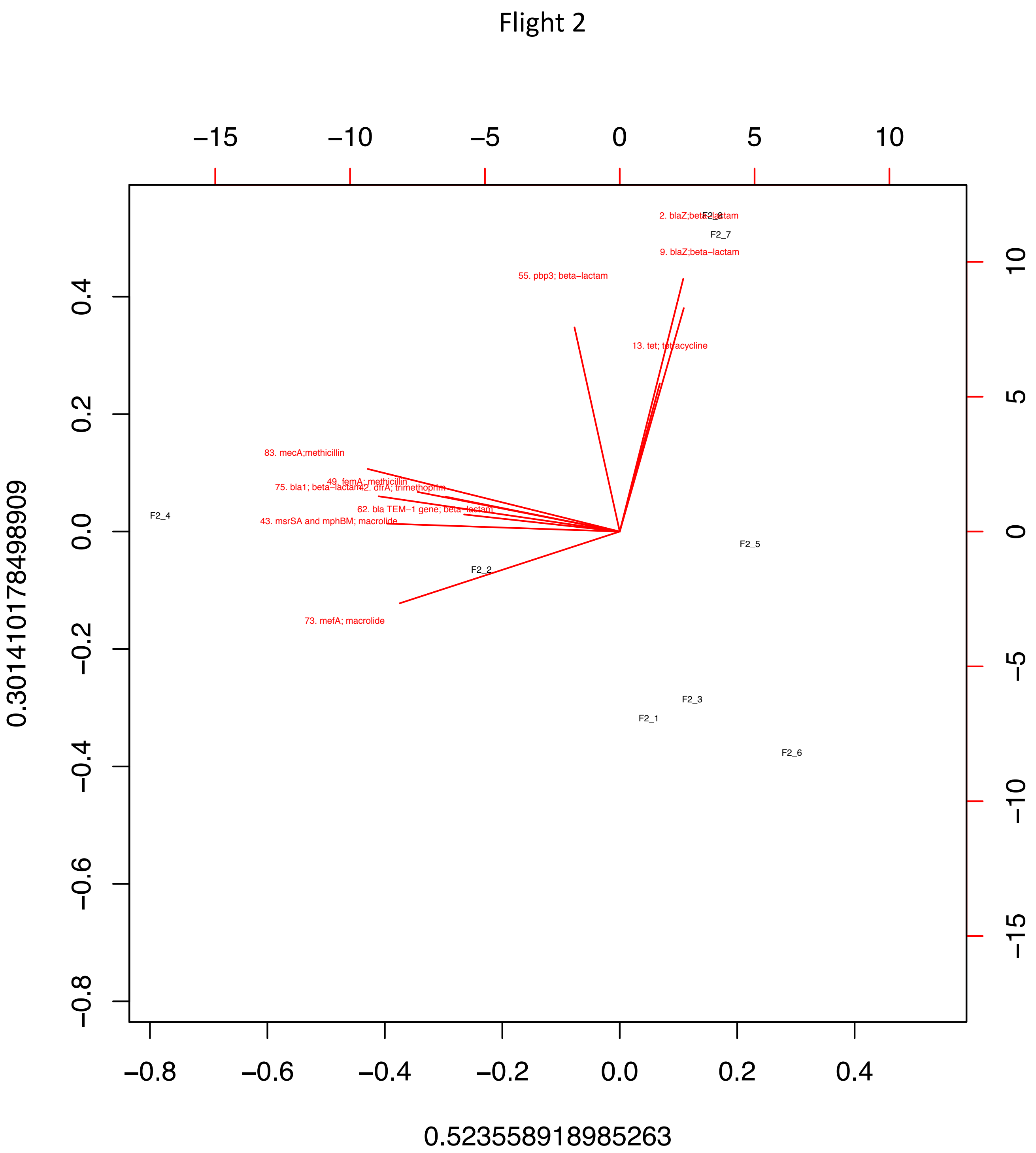

Flight 2
−15
−10
−5
0
5
10
2. blaZ;beta−lactam
F2_8
F2_7
9. blaZ;beta−lactam
55. pbp3; beta−lactam
13. tet; tetracycline
83. mecA;methicillin
49. femA; methicillin
75. bla1; beta−lactam
42. dfrA; trimethoprim
62. bla TEM−1 gene; beta−lactam
43. msrSA and mphBM; macrolide
F2_4
F2_5
F2_2
73. mefA; macrolide
F2_3
F2_1
F2_6
0.4
0.2
0.0
−0.2
−0.4
−0.6
−0.8
0.301410178498909
−0.8
−0.6
−0.4
−0.2
0.0
0.2
0.4
0.523558918985263

Fig S1C

Flight 3

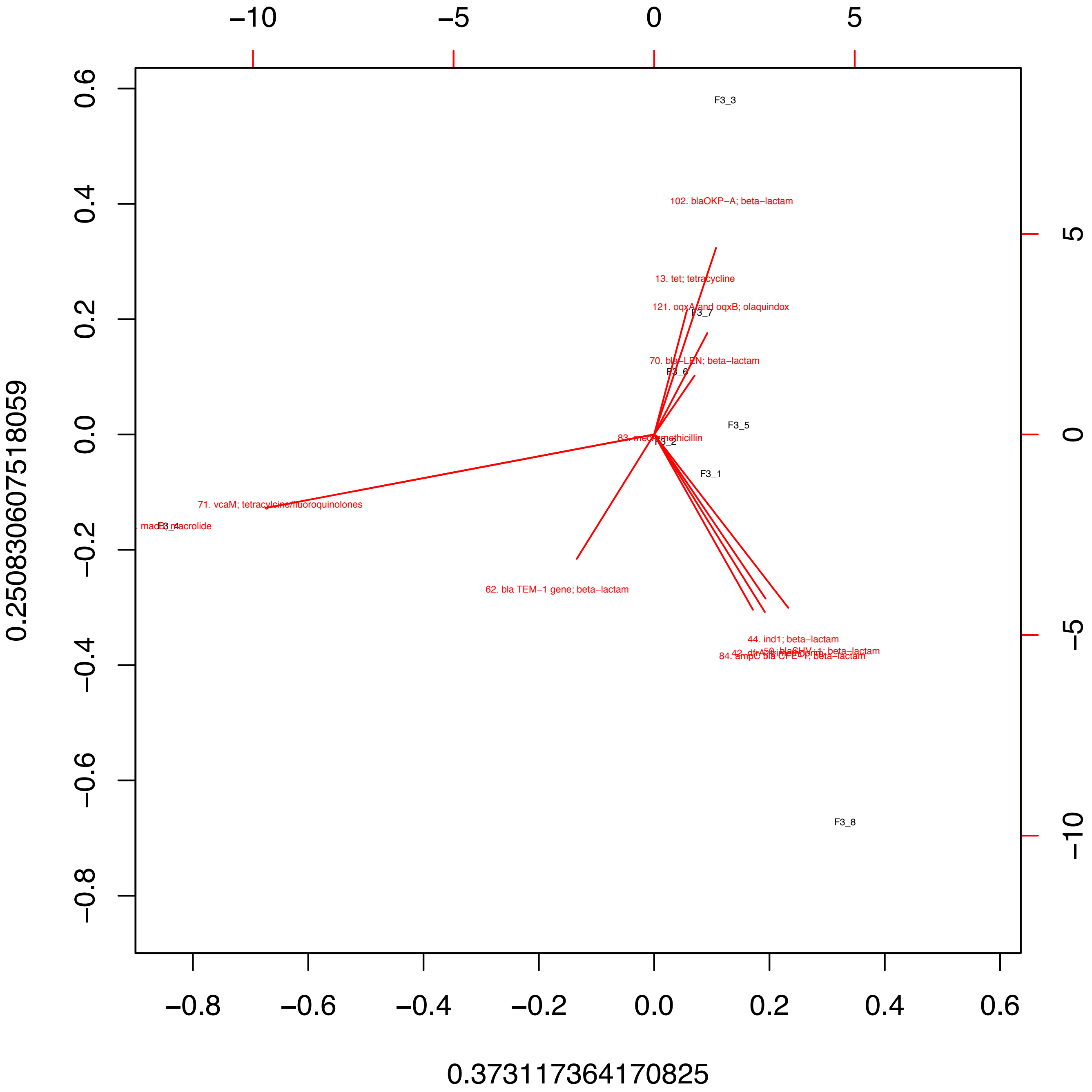

Figure S1. **Compositional biplot showing the variance amongst samples**.

Each sample (represented by the sample name in black) is plotted on this 2-D plane showing the variance amongst samples. Samples that are closer together are more similar in AMR gene composition (i.e. contain similar sets of genes at similar ratios) then those farther away on the plot. The arrows in red represent the AMR gene variance. The longer the arrow is from the end point to the center, the more variable this gene is amongst the samples and can help explain compositional differences amongst samples. The closer a gene is to the center, the least variable it is amongst samples. For example, for Flight 1: F1_1, F1_6, F1_8, F1_5, F1_3, F1_7 are similar in composition and the ratio of *bla* TEM-1, *mefA*, *dfrA* and *bla* SHV-1 compared to other genes is similar in these samples compared to others. (A) Flight 1 (B) Flight 2 (C) Flight 3.
